# Role of ABCA7 loss-of-function variant in Alzheimer's disease: a replication study in European–Americans

**DOI:** 10.1186/s13195-015-0154-x

**Published:** 2015-12-10

**Authors:** Jorge L. Del-Aguila, Maria Victoria Fernández, Jessica Jimenez, Kathleen Black, Shengmei Ma, Yuetiva Deming, David Carrell, Ben Saef, Alzheimer’s Disease Neuroimaging Initiative, Bill Howells, John Budde, Carlos Cruchaga

**Affiliations:** Department of Psychiatry, Washington University School of Medicine, 660 South Euclid Avenue B8134, St. Louis, MO 63110 USA; The Hope Center for Neurological Disorders, BJC Institute of Health, 425 S. Euclid Ave, St. Louis, MO 63110 USA

## Abstract

**Introduction:**

A recent study found a significant increase of *ABCA7* loss-of-function variants in Alzheimer’s disease (AD) cases compared to controls. Some variants were located on noncoding regions, but it was demonstrated that they affect splicing. Here, we try to replicate the association between AD risk and ABCA7 loss-of-function variants at both the single-variant and gene level in a large and well-characterized European American dataset.

**Methods:**

We genotyped the *GWAS* common variant and four rare variants previously reported for *ABCA7* in 3476 European–Americans.

**Results:**

We were not able to replicate the association at the single-variant level, likely due to a lower effect size on the European American population which led to limited statistical power. However, we did replicate the association at the gene level; we found a significant enrichment of *ABCA7* loss-of-function variants in AD cases compared to controls (*P* = 0.0388; odds ratio =1.54). We also confirmed that the association of the loss-of-function variants is independent of the previously reported genome-wide association study signal.

**Conclusions:**

Although the effect size for the association of *ABCA7* loss-of-function variants with AD risk is lower in our study (odds ratio = 1.54) compared to the original report (odds ratio = 2.2), the replication of the findings of the original report provides a stronger foundation for future functional applications. The data indicate that different independent signals that modify risk for complex traits may exist on the same locus. Additionally, our results suggest that replication of rare-variant studies should be performed at the gene level rather than focusing on a single variant.

## Introduction

A recent study found that loss-of-function variants in *ABCA7* (ATP-binding cassette transporter A7) confer greater risk for Alzheimer’s disease (AD) [1]. Steinberg et al. [1] analyzed sequence, genome-wide association study (GWAS), and linkage data from 3419 individuals with AD and 151,805 controls from Iceland. Gene-based analyses, including nonsense, missense, frameshift splice-site variants and canonical splice-site variants (‘loss-of-function’), identified *ABCA7* as the most significant gene (odds ratio (OR) = 2.12, *P* = 2.2 × 10^–13^) for AD. This association was mainly driven by a single splice-site variant, rs200538373 (OR = 4.47, *P* = 3.4 × 10^–7^), although other coding variants and splice variants were also found. This association was replicated at the gene level by genotyping the loss-of-function variants in more than 6500 AD cases and controls from four independent datasets (OR = 1.73, *P* = 0.0056). Interestingly, the OR for the variant (rs200538373) that led the association on the discovery series was in the opposite direction in the replication dataset (OR = 0.93). Additionally, none of these loss-of-function variants were in linkage disequilibrium (LD) with the *ABCA7* common variant identified by GWAS [2], suggesting that there are multiple and independent mechanisms throughout the *ABCA7* region that increase risk for AD. Based on these results, we tried to replicate the association of the *ABCA7* loss-of-function variants in a large cohort of European–Americans.

## Methods

A total of 1776 AD cases and 1700 controls were selected from the Alzheimer's Disease Neuroimaging Initiative (ADNI), the Charles F. and Joanne Knight Alzheimer's Disease Research Center (Knight-ADRC) and the National Institute on Aging Genetics Initiative for Late-Onset Alzheimer’s Disease (NIALOAD) [3, 4]. The Institutional Review Board at the Washington University School of Medicine in Saint Louis approved the study. Research was carried out in accordance with the approved protocol. Written informed consent was obtained from participants and their family members by the Clinical Core of the Knight-ADRC. The approval number for the Knight-ADRC Genetics Core family studies is 93-0006.

In the original study, Steinberg et al. [1] genotyped the *ABCA7* variants on 6681 European non-Icelandic individuals and only four variants were polymorphic (Supplementary Table 5 from Steinberg et al. [1]). Therefore, we decided to genotype the variants that were found to be polymorphic on the non-Icelandic population together with the GWAS common variant. The *ABCA7* GWAS common variant (rs4147929) as well as the loss-of-function variants (p.Glu709Alafs*86, p.Leu1403Argfs*7, rs200538373, and rs113809142) reported by Steinberg et al. [1] were genotyped in our dataset using competitive allele-specific polymerase chain reaction KASPar genotyping technologies as described previously [3, 4].

Allelic frequencies, Hardy-Weinberg equilibrium and single-variant association tests were performed with PLINK-1.9 [5]. For gene level analysis, burden analyses were performed using a combined multivariate and collapsing (CMC) test [6]. Age, gender, and principal component factors (PCs) were included in all association tests as covariates.

## Results and discussion

In our dataset, the common variant rs4147929[A] was significantly associated with AD risk (Table 1; OR = 1.162, *P* = 0.022), suggesting that we have enough power to replicate known associations for common variants. All the genotyped loss-of-function variants were polymorphic in our dataset with similar frequencies to those reported in the European non-Icelandic datasets. As initially reported, none of the genotyped variants were in LD with the common variant rs4147929, allowing for independent analysis. In our dataset, all rare variants were more frequent in AD cases than in controls with an OR ranging from 1.2 to 1.7; however, none of these values were statistically significant on their own (Table 1; *P* > 0.1).

**Table 1:** Association of *ABCA7* variants with Alzheimer’s disease in studied groups based on Fisher’s association test, including age, gender, and PC as covariates. Gene-based analysis was conducted with CMC collapsing method

rsID/position^a^	MAF		OR	*P*
	Cases	Controls	(95 % CI)	
rs4147929	0.179	0.1581	**1.162**	**0.022**
(GWAS SNP)			**(1.02–1.31)**	
19:998507^a^	0.006	0.0037	1.735	0.121
p.Glu709AlafsX86			(0.54**–**3.04)	
19:1006907^a^	0.003	0.0027	1.285	0.569
p.Leu1403ArgfsX7			(0.76**–**3.21)	
rs113809142	0.002	0.0012	1.695	0.451
c.4416+2T>G			(0.34**–**5.91)	
rs200538373	0.009	0.0076	1.231	0.476
c.5570+5G>C			(0.68**–**2.61)	
Loss-of-function	0.016	0.0107	**1.549**	**0.038**
(All low frequency variants)			**(1.02–2.34)**	

^a^NCBI Build 36

MAF

Values shown in bold are significant at the *P* < 0.05 level

*CI* Confidence interval, *CMC* Combined multivariate and collapsing, *GWAS* Genome-wide association study, *MAF* Minor allele frequency, *OR* Odds ratio, *PC* Principal component factors, *SNP* Single Nucleotide Polymorphism

Although we had statistical power to replicate the association of rs113809142 with AD risk based on the effect size reported in the Icelandic population (OR = 4.42, power = 0.887), we did not have enough power to replicate the single-variant analyses based on the minor allele frequency (MAF) and effect sizes reported on the European non-Icelandic dataset. There is some debate concerning what is the best approach to replicate the association of rare variants with complex traits [7–11]. It is clear that the MAF for these low-frequency variants varies widely, not only among populations, but also within populations [10–12]. If the cases and controls are not very well matched for local genetic background, the study can produce false-positive or false-negative results [7, 11, 13]. Additionally, it is possible that a specific variant is not found, or found in an extremely low frequency in a specific population; as with the case of the *TREM2* R47H variant in Asian [14, 15] or African–American populations. Although the association of the R47H variant with AD risk has been widely replicated in European–Americans, no significant association is found in Asian or African–American populations, because the MAF for this variant (R47H) is extremely low. However, other variants in the same gene could increase risk for diseases in these populations. Steinberg et al. [1] failed to replicate the association of the rs113809142 in the European non-Icelandic population, but they were able to replicate the association at the gene level. These results support the notion that different (local) populations have varying genetic make-ups, and therefore single-variant analyses may not be the best approach for replicating these studies. This hypothesis is also supported by recent studies from Jin et al. [3, 15] in which deep resequencing of *TREM2* was performed on European–Americans and African–Americans; different variants were found in each population, and the variants in common presented very different MAFs and ORs. However, in both cases, the gene-based analyses supported the association of *TREM2* with AD risk.

For this reason, we decided to perform a gene-based analysis for all the reported polymorphic *ABCA7* loss-of-function variants. Since all variants presented the same direction of effect, we performed a CMC test. In our dataset, we found a significant enrichment of *ABCA7* loss-of-function variants in AD cases compared to controls (*P* = 0.0388; OR = 1.54). Therefore, despite none of the individual loss-of-function variants reported a significant association with *ABCA7* in this study, we were able to replicate two independent signals of the correlation of the *ABCA7* gene with AD: the common variant and the aggregation of the loss-of-function variants.

The point estimate for the OR in this study for the gene-based analysis is slightly lower than the reported OR for the Icelandic population (OR = 2.12) or the European non-Icelandic replication datasets (OR = 1.73), although the 95 % confidence interval in this study (1.02–2.34) includes both the Icelandic OR and the European non-Icelandic OR published by Steinberg et al. [1]. The current findings and those of the European non-Icelandic population support a possible "winner's curse" for the Icelandic discovery. Our sample size was smaller than both of the discovery series. Additionally, a proper gene-based replication would entail resequencing the candidate region to identify novel functional variants, and not just genotyping the reported variants; therefore, the real OR for the *ABCA7* loss-of-function variants remains to be determined. Despite these limitations, we were able to replicate the original report. Our data also indicate that the gene-based association of these loss-of-function variants is independent of the GWAS variant, and that the aggregate effect of these variants is larger than that of the common variant alone.

Our study validates the role of noncoding loss-of-function *ABCA7* variants in AD risk. Other population-specific independent variants with similar loss-of-function effects may contribute to AD risk or other complex traits. Supporting this hypothesis, a recent study has reported on an additional intronic low-frequency variant of *ABCA7* (rs78117248; OR = 2.07, *P* = 0.0016) that increases risk for AD, also independently of the common variant. Together, these results suggest that different and independent variants modify risk for complex diseases by different mechanisms existing on the same locus [16, 17]. Other genes will also harbor rare variants increasing risk for AD, independently of the GWAS hits [17].

## Conclusions

In summary, our study replicates the association of *ABCA7* loss-of-function variants with AD risk, and highlights the necessity of performing gene-based, rather than single-variant analyses to replicate the association in this type of studies. Our study also confirms that there is high variability in the MAF of low-frequency variants within a population, so matching cases and controls for genetic background is a key step to avoiding false negatives or positives.

## Abbreviations

*ABCA7*: ATC-binding cassette, subfamily A (ABC1), member 7
AD: Alzheimer’s disease
ADNI: Alzheimer's Disease Neuroimaging Initiative
ADRC: Alzheimer's Disease Research Center
CMC: Combined multivariate and collapsing
GWAS: Genome-wide association study
LD: Linkage disequilibrium
MAF: Minor allele frequency
NIALOAD: National Institute on Aging Genetics Initiative for Late-Onset Alzheimer’s Disease
OR: Odds ratio
PC: Principal component factors
